# Electroacupuncture Improves Gastric Emptying in Critically Ill Neurosurgical Patients: A Pilot Study

**DOI:** 10.1155/2017/1892161

**Published:** 2017-09-20

**Authors:** Man-Ling Kao, Yao-Li Chen, Shu-Ching Lee, Sung-Yen Huang, Ping-Yi Lin

**Affiliations:** ^1^Department of Surgical Critical Care, Changhua Christian Hospital, Changhua, Taiwan; ^2^Department of General Surgery, Changhua Christian Hospital, Changhua, Taiwan; ^3^School of Medicine, Kaohsiung Medical University, Kaohsiung, Taiwan; ^4^Department of Chinese Medicine, Changhua Christian Hospital, Changhua, Taiwan; ^5^Department of Medical Research, China Medical University Hospital, Taichung, Taiwan; ^6^Transplant Medicine & Surgery Research Centre, Changhua Christian Hospital, Changhua, Taiwan

## Abstract

**Objective:**

To compare the efficacy of combined electroacupuncture and metoclopramide treatment with that of metoclopramide only in improving gastric emptying in critically ill neurosurgical patients.

**Methods:**

In this prospective case-control pilot study, a total of 16 adult critically ill mechanically ventilated patients who were treated in the surgical intensive care unit were enrolled. Electrical stimulation was applied to 4 pairs of points (maximum intensity < 9.8 mA at 2 Hz). Patients in the control group received standard treatment with intravenous metoclopramide only. Patients in the experimental group received intravenous metoclopramide plus electroacupuncture treatment once daily for 6 consecutive days.

**Results:**

Gastric residual volume in the experimental group (*n* = 7) reduced gradually until the fourth day after treatment with electroacupuncture combined with routine metoclopramide administration. Beginning on the fourth day, residual volume was maintained at less than 200 ml per day for the following two days. In the control group (*n* = 9), there was a gradual reduction in residual volume during the first four days followed by a rebounding increase over the next two days.

**Conclusions:**

Electroacupuncture combined with intravenous metoclopramide is a more effective treatment for gastric emptying than metoclopramide alone in adult critically ill patients with impaired brain function.

## 1. Introduction

Inadequate nutrition is an important problem in critically ill patients. Enteral feeding is the preferred method because it not only offers nutritional advantages but also contributes to bowel flora maintenance, reduces infection risks, and avoids the adverse outcomes of parenteral nutrition [1, 2]. Unfortunately, attempted early enteral nutrition is often complicated by delayed gastric emptying, as indicated by large gastric residual volumes and gastroesophageal reflux, especially in patients who have sustained trauma or undergone surgery [3].

Gastrointestinal motility disorders are common in the critically ill and can be caused by a number of factors including the presenting injury or illness, preexisting comorbidities, age, and the administration of drugs during admission [4]. Delayed gastric emptying is commonly encountered in the intensive care unit (ICU), occurring in up to 60% of all ICU patients and in the vast majority (80%) of patients suffering from cerebral hypertension due to skull or brain trauma. Gastrointestinal motility disorders can lead to regurgitation or vomiting, which can lead to aspiration of gastric contents resulting in aspiration pneumonia [5]. Enteral feeding intolerance is associated with malnourishment, fewer ventilator-free days, increased ICU stay, and increased mortality [3].

Metoclopramide is a commonly used prokinetic drug in the ICU. The typical dosage in the ICU setting is 10 mg 4 times daily, although 10 mg 3 times daily is also used. In patients with renal failure, the dose should be reduced by 50% when creatinine clearance is 10 to 50 ml/minute [3]. Metoclopramide increases antral and small intestinal motility but has limited effect in the distal small bowel. Intravenous metoclopramide is frequently used to treat delayed gastric emptying, but no benefit has been shown when metoclopramide is delivered enterally or to those with traumatic brain injury [6]. Gastric residual volume (GRV) measurement has been incorporated into specific guidelines for gastrointestinal function. Standards for the management of GRV, however, are not evidence-based [2]. However, it has been reported that high GRV may increase the risk for aspiration in critical illness patients [7]. Thus, the most frequently threshold levels for interrupting feedings are 200 ml and 250 ml to minimize the risk of aspiration [5]. Acupuncture involves inserting the tips of thin, stainless steel needles through the skin at specific points (acupoints). The procedure can be accomplished by manual manipulation or electrical stimulation (elecroacupuncture) [8]. Electroacupuncture is achieved by attaching the acupuncture needles to an electrical pulse generator and stimulating the acupoints with electrical pulses [9]. The effects of acupuncture, including electroacupuncture, may depend on the selected acupoints, the combination of acupoints, and the intensity of stimulation [10]. Acupuncture has been shown to be helpful in restoring gastrointestinal barrier injury by regulating the neuron-endocrine-immune system and antagonizing the inflammatory response [8]. Acupuncture has been demonstrated to promote gastric peristalsis in subjects with low initial gastric motility and suppress peristalsis in those with active initial motility [11].

In this prospective, case-control study, we evaluated the efficacy of combined electroacupuncture/metoclopramide treatment with that of metoclopramide only in improving gastric emptying in critically ill neurosurgical patients.

## 2. Methods

### 2.1. Study Design and Patients

The study was a prospective case-control pilot study comparing the effect of electroacupuncture and a prokinetic drug on gastric emptying in adult critically ill neurosurgical patients. Study subjects consist of patients with first-ever stoke due to spontaneous intracerebral hemorrhage, spontaneous subarachnoid hemorrhage, spontaneous cerebellar hemorrhage, or a recent head injury who presented with delayed gastric emptying and poor enteral feeding for more than 6 days. Delayed gastric emptying was defined as a gastric residual volume of at least 300 ml per 24 hours measured via a nasogastric tube after admission to the surgical intensive care unit. Exclusion criteria included age younger than 18 years or older than 80 years, history of abdominal surgery or abdominal trauma, recent intra-abdominal inflammation or infection, bowel obstruction, recent gastrointestinal bleeding, upper or lower extremity deformity with local skin infections or skin defects, diabetes mellitus, hepatic or renal failure, and pregnancy. A total of 16 adult critically ill mechanically ventilated patients who were treated in the surgical intensive care unit at the Changhua Christian Hospital during the period April 2014 to May 2015 fulfilled the inclusion criteria and were enrolled in the study. A written informed consent was obtained from a relative legally authorized. The protocol was approved by the Institutional Review Board of the Changhua Christian Hospital and the study was conducted according to the principles of the Declaration of Helsinki. In this study, simple randomization method was used. Patients were randomly assigned either to receive standard treatment with intravenous metoclopramide only (control group, *n* = 7) or to receive intravenous metoclopramide plus electroacupuncture intervention (experimental group, *n* = 9).

### 2.2. Prokinetic Drug Administration

Patients in both groups received intravenous metoclopramide 10 mg every 8 hours for 6 consecutive days. None of the patients received sedative agents or muscle relaxants during treatment.

### 2.3. Electroacupuncture Intervention

**Figure 1:**
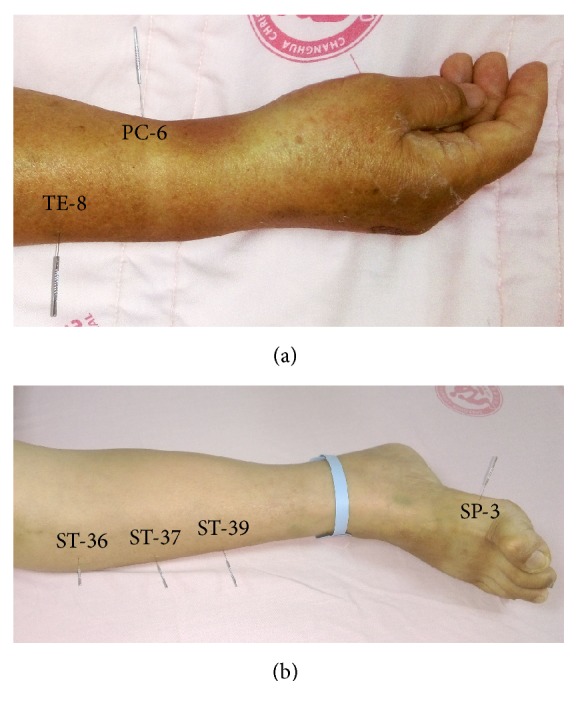
Acupuncture points (a) PC-6 and TE-8; (b) ST-36, ST-37, ST-39, and SP-3.

Electroacupuncture was performed by a licensed practitioner of Traditional Chinese Medicine with 12 years of experience. The acupoints selected in this study are known to regulate gastrointestinal function and included the bilateral PC-6 (Neiguan), TE-8 (Sanyanglou), ST-36 (Zusanli), ST-37 (Shangjuxu), ST-39 (Xiajuxu), and SP-3 (Taibai) acupoints (Figure 1). Of those acupoints, PC-6, TE-8, ST-36, and SP-3 belong to source and lower sea points which can improve digestive function and increase gastrointestinal motility (Table 1) [12–14]. Single-use, sterilized, disposable stainless steel acupuncture needles measuring 0.30 mm in diameter and 40 mm in length were used in all procedures (An Chi Handy Disposable Acupuncture Needle, Inc., Taipei, Taiwan). Acupuncture was performed with the patient lying in supine position. After the skin was sterilized with 70% alcohol, the needles were inserted perpendicularly at an approximate depth of 1 cun and manipulated until the acupuncturist felt a tight sensation around the needled acupuncture points. Electrical stimulation was provided by an electroacupuncture stimulator (Z-7023, ZMI Multichannel TENS, Taiwan) and applied at a frequency of 2 Hz and with a proper current intensity when the needles showed a slight twitch (maximum intensity < 9.8 mA). Electrical stimulation was applied to 4 pairs of points, including bilateral PC-6 and TE-8 and bilateral ST-36 and SP-3 [12–14]. Each electroacupuncture treatment lasted approximately 20 min. All patients in the experimental group received electroacupuncture treatment once daily for 6 consecutive days.

### 2.4. Nasogastric Tube Feeding and Data Collection

All patients received gastric enteral feeding via a 14 Fr polyvinylchloride nasogastric tube that had been inserted transnasally and placed 30 cm above the gastric level. On the first day after admission, only crystalloid infusions were given. Enteral feeding was initiated on the second day with all patients in the Semi-Fowler's position with the head of the bed at 30° to 60°. Administration of standard high-protein formula was continuous (10–20 ml/h) and controlled by a feeding pump delivery system. Total daily calories were increased gradually to 30 kcal/kg body weight on the following day. The enteral feeding was temporarily withheld if gastric residual volume was greater than 500 ml or vomiting episodes occurred. All data were obtained by the primary investigators and by trained nurses in the SICU (surgical intensive care unit). Gastric residual volume was measured in milliliters and checked every 4 hours using a 50 ml syringe to aspirate the residual gastric contents as completely as possible. The residual gastric content was reintroduced to the patient if the volume aspirated was less than 500 ml. Measurement of the aspirated and returned amounts and total daily intake were recorded by the nursing staff.

### 2.5. Statistical Analysis

The chi-square test or Fisher's exact test was used for categorical comparisons of data. The Mann–Whitney *U* test was used to compare continuous variables between patients who received combined treatment and those who received metoclopramide only. A *p* value of less than 0.05 was considered to indicate statistical significance. All statistical analyses were performed using the statistical package SPSS for Windows (Version 17.0, SPSS Inc.; Chicago, IL, USA).

## 3. Results

The 16 patients are comprised of 2 women and 14 men, and the overall mean age was 47.4 years (range, 20–78 years). None of the patients in this study had a history of abdominal lesions or abdominal surgery; therefore, we can rule out most causes of digestive problems, such as intestinal ischemia, intestinal obstruction, and adhesion ileus. The baseline characteristics of both groups are shown in Table 2. There were no significant differences in mean age, BMI (body mass index), total GCS (Glasgow coma scale) score, or APACHE (acute physiology and chronic health evaluation) II scores between the two groups. Hypertension was present in 3 of the 16 patients. The indications for admission included spontaneous cerebral hemorrhage in 5 patients and head trauma in 11 patients. All patients were mechanically ventilated.

**Figure 2:**
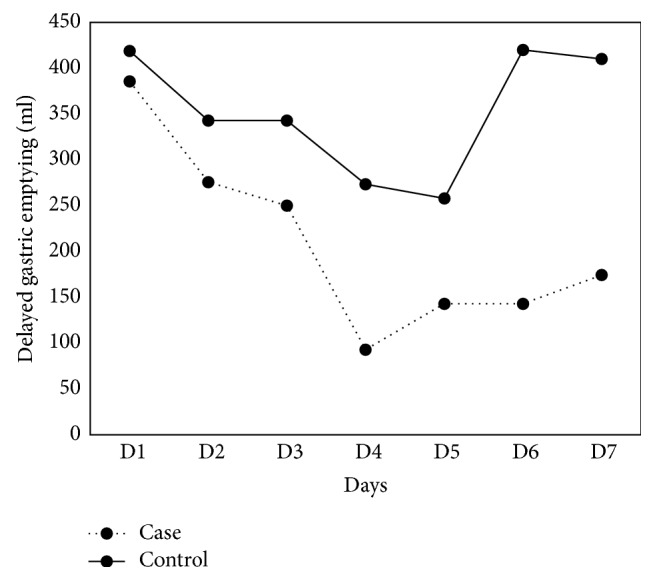
Results of electroacupuncture on gastric emptying.

The effects of electroacupuncture on gastric emptying are shown in Figure 2. Gastric residual volume in the experimental group reduced gradually until the fourth day after treatment with electroacupuncture and routine metoclopramide administration. On the fifth day, the gastric residual volume was less than 200 ml per day. In the control group, gastric residual volume reduced initially; however, there was a rebounding increase with time during the next few days. There were no significant adverse reactions during treatment in either group.

## 4. Discussion

In the control group, the routine use of metoclopramide alone resulted in a short-term decrease in gastric residual volume. Prokinetic agents are used prophylactically to improve feeding efficacy and prevent vomiting and ventilator-associated pneumonia in ICU patients. Metoclopramide as an agent to improve gastric emptying should be applied only for short periods (days) in the ICU so as to limit the risk of adverse neurological and cardiac reactions. Over time, treatment becomes less effective, with treatment failure due to tachyphylaxis occurring after a few days. Desensitisation, downregulation, and endocytosis of neurohumoral receptors have been proposed as mechanisms underlying the occurrence of tachyphylaxis [15].

In the experimental group, receipt of electroacupuncture and metoclopramide resulted in a gradual reduction in gastric residual volume, eventually reaching a valley point of approximately 200 ml per day. Many studies have reported that acupuncture treatment contributes to the maintenance of the biochemical balance of the central nervous system [10]. Li et al. reported that electroacupuncture stimulated the release of neurotransmitters and endogenous substances [16]. Acupuncture has also been shown to cause multiple biologic responses in many structures that are mediated mainly through sensory neurons within the central nervous system. These responses may be partially explained by the fact that acupuncture can induce long-lasting changes in neuronal gene expression leading to persistent neuronal input modulation [17].

The basic theory of acupuncture is that the insertion and manipulation of a needle at a particular point or points along a meridian related to an impaired organ stimulate energy flow, restore a proper energy balance, and normalize the functions of the organ. A review by Ouyang and Chen found that the results of electroacupuncture are often more consistent and reproducible than nonelectroacupuncture [9].

Studies have also shown that needle retention for 30 minutes is more effective than the 5-minute retention period commonly used in clinical practice [18]. In a recent functional magnetic resonance imaging study of the human brain demonstrated that manual acupuncture at the ST-36 acupoint modulated neural activity at multiple levels in the cerebrocerebellar and limbic systems [8]. In addition, acupuncture at the PC-6 acupoint has been shown to selectively activate the left superior frontal gyrus, anterior cingulated gyrus, and dorsomedial nucleus of the thalamus in healthy humans [19]. The current study is a pilot study and limited by the small patient numbers. Further studies are needed to confirm our results.

## 5. Conclusion

Electroacupuncture combined with intravenous metoclopramide is an effective treatment for delayed gastric emptying in critically ill adult patients with impaired brain function.

## Figures and Tables

**Table 1:** Acupuncture points.

Point	Meridian	Characteristic	Location
TE-8 (Sanyanglou)	Triple energizer meridian		4 cun proximal to the dorsal wrist crease, midpoint of the interosseous space between the radius and the ulna

PC-6 (Neiguan)	Pericardium meridian	Connecting point of pericardium meridian	2 cun proximal to the palmar wrist crease, between the tendons of the palmaris longus and the flexor carpi radialis

ST-36 (Zusanli)	Stomach meridian	Lower sea points of stomach meridian	3 cun below ST 35, one finger-breadth from anterior crest of tibia

ST-37 (Shangjuxu)	Stomach meridian	Lower sea points of large intestine meridian	6 cun below ST 35, one finger-breadth from anterior crest of tibia

ST-39 (Xiajuxu)	Stomach meridian	lower sea points of small intestine meridian	9 cun below ST 35, one finger-breadth from anterior crest of tibia

SP-3 (Taibai)	Spleen meridian	Source point of spleen meridian	On the medial aspect of the foot, in the depression proximal to the first metatarsophalangeal joint, at the border between the red and white flesh

**Table 2:** Baseline patient characteristics.

	Study group	Control group	*p*	Total
	*n* = 7	*n* = 9		*N* = 16
Age			0.40	
Mean (SD)	51 (17.1)	44.7 (22.3)		47.4 (19.8)
Gender			1.00	
Female	1 (14.3)	1 (11.1)		2 (12.5)
Male	6 (85.7)	8 (88.9)		14 (87.5)
Body mass index			0.12	
Mean (SD)	25.4 (5.5)	21.3 (3.31)		23.1 (4.7)
Glasgow coma scale			0.63	
Mean (SD)	5.4 (2.2)	7 (3.9)		6.3 (3.3)
Diabetes			—	
Yes	0 (0)	0 (0)		0 (0)
No	7 (100)	9 (100)		16 (100)
Hypertension			0.06	
Yes	3 (42.9)	0 (0)		3 (18.8)
No	4 (57.1)	9 (100)		13 (81.2)
APACHE II			0.45	
Mean (SD)	19.7 (4.8)	16.9 (6.6)		18.1 (5.8)
Ventilator support			—	
Yes	7 (100)	9 (100)		16 (100)
No	0 (0)	0 (0)		0 (0)
Intracranial pressure monitor			—	
Yes	7 (100)	8 (88.9)	1.00	15 (93.8)
No	0 (0)	1 (11.1)		1 (6.2)

## Abbreviations

ICU: Intensive care unit
GRV: Gastric residual volume
SICU: Surgical intensive care unit
BMI: Body mass index
GCS: Glasgow coma scale
APACHE II: Acute physiology and chronic health evaluation.

## Additional Points

*Availability of Data and Material*. The data are available from the authors.
